# Update on human papilloma virus - part I: epidemiology, pathogenesis, and clinical spectrum^☆^^☆☆^

**DOI:** 10.1016/j.abd.2020.11.003

**Published:** 2020-12-10

**Authors:** Geraldo Magela Magalhães, Érica Cristina Vieira, Lucas Campos Garcia, Maria de Lourdes Ribeiro De Carvalho-Leite, Antônio Carlos Martins Guedes, Marcelo Grossi Araújo

**Affiliations:** a Department of Internal Medicine, Faculty of Medicine, Universidade Federal de Minas Gerais, Belo Horizonte, MG, Brazil; b Dermatology Service, Hospital das Clínicas, Universidade Federal de Minas Gerais, Belo Horizonte, MG, Brazil; c Minas Gerais State Department of Health, Belo Horizonte, MG, Brazil

**Keywords:** Papillomaviridae, Tumor virus infections, Tumor virus infections/complications, Tumor virus infections/diagnosis, Tumor virus infections/epidemiology

## Abstract

Infection with human papilloma virus (HPV) is related to a great number of cutaneous and mucosal manifestations. The spectrum of HPV ranges from inapparent infections, through various clinical benign presentations including cutaneous and mucosal disease, to malignant and premalignant conditions. New HPV types are currently described in the literature; many of them are characterized as high-risk types due to their oncogenic potential. Knowledge regarding their epidemiology and pathogenesis is important to understand not only infection and disease processes, but also to formulate the clinical and laboratory basis for diagnosis, therapeutics, and prophylactic measures. This non-systematic review aims to discuss and to update those aspects, with an emphasis on relevant topics for dermatologists. HPV infection and related diseases in the Brazilian scenario are highlighted, including common dermatologic conditions seen at clinics as well as the condition of a public health problem as a sexually transmitted infection. The oncogenicity of the virus and the variety of clinical outcomes – especially in the immunocompromised individuals – are addressed.

## Introduction

The high prevalence of infection by the human papilloma virus (HPV) and its relationship with diseases, ranging from benign conditions in the skin and mucous membranes to the most frequent sexually transmitted infection (STI), indicate its importance in the public health scenario and the role of the dermatologist as one of the professionals trained to diagnose and treat many of these diseases.^1^ Among the more than 400 types of papillomavirus (PV) described, 218 are related to infections in humans.^2^ Its oncogenic potential is well established in several types of neoplasms and has been amplified by situations of immunosuppression in increasingly large segments of the population, compromising the survival and quality of life of the affected individuals^3^, ^4^ Prophylactic vaccines targeting various types of HPVs that cause benign or malignant lesions on mucous membranes are available in several countries worldwide, unfortunately still with suboptimal coverage.^5^

In turn, advances in treatment are incorporated more slowly into the therapeutic arsenal, showing the importance of early diagnosis and health education as preventive measures. In this non-systematic review, the authors searched for articles that would aid in updating and discussing the aspects of epidemiology, pathogenesis, and main clinical manifestations related to HPV.

## Epidemiology

Currently, approximately 218 types of HPV have been isolated and identified as causing infections in humans.^2^ Of these, 45 infect the genital tract, while the others will cause skin disease.^6^ Alpha HPVs produce clinically visible lesions on mucous membranes and skin, while beta and gamma HPVs are mainly responsible for persistent subclinical skin lesions secondary to infections acquired early in childhood.^7^ The infection occurs predominantly *via* direct contact, although skin lesions can be transmitted indirectly, *via* contaminated surfaces.^8^ Microtraumas expose the basal layer keratinocytes and facilitate contagion.^7^

Cutaneous warts (CWs) related to non-sexually transmitted HPV types have completely different epidemiological characteristics and are mainly caused by types 1, 2, 4, 27, and 57.^9^, ^10^ Although these are the most prevalent types in several studies, the proportion of individuals affected by each of them is quite variable. Some authors speculate that the differences are due to socio-geographical variations.^9^ A study conducted in Dutch schools found CWs in approximately one-third of the participants, with the prevalence increasing with age, from about 15% at 4 years to about 44% at 11 years, with no difference between genders.^10^ Infection with HPV 1 affects a younger age group, more often on the plantar region, with fewer and smaller lesions, while HPV 2 is responsible for the second peak, in the second and third decades of life.^9^, ^10^

The main risk factors found in school patients were close family members with CWs and families with multiple children. Transmission *via* public toilets and changing rooms was not statistically relevant in that study.^10^ Autoinoculation is also described, especially in the presence of lesions in the fingers. The prevalence of CWs in the general population is estimated at around 5% and is the result of continuous and repeated infection by multiple types.^11^

Genital HPV lesion is the most prevalent STI in the world.^12^ In Brazil, a recent systematic review carried out by Colpani et al. observed the following prevalences of HPV in mucosal lesions: 36.21% in the penile region; 25.68% in the anal region; 24.11% in the uterine cervical region; and 11.89% in the oral region.^1^ The study included more than 50,000 participants of all ages; the prevalence was higher than that of Central America (13%) and Western Europe (9%).^1^ Another multicenter, community-based Brazilian study of unvaccinated young people found high-risk HPV (HR-HPVs) in 35.2% of individuals, with a predominance of women.^13^ However, when analyzing sexually active young people, this percentage reached 53.5% for all types of HPV, regardless of sex.^13^ It is estimated that 75% of sexually active adults will have at least one HPV infection in their lifetime. The most frequently involved are types 6 and 11, present in about 80% of oral lesions and more than 90% of genital lesions.^12^ Risk factors include number of partners and age at first sexual intercourse, immunosuppression (including HIV), and the presence of other STIs, such as herpes simplex. The relationship with smoking and the use of oral contraceptives remains controversial.^12^ Contagion can occur through any form of sexual contact, even without penetration.^12^ The incidence of anal infection is high among men who have sex with men (MSM).^14^ Lesions in men appear to be less persistent than in women.^15^

The peak incidence of infection occurs about 10 years after sexarche, usually between 24 and 30 years of age for both sexes. Although the prevalence decreases with age, studies show a second late peak of incidence in women in the fifth decade.^12^, ^13^ The episodes of clinical manifestation have an mean duration of two and a half months. For each of these, a mean of three medical consultations are held.^16^

In the anogenital region, HPV – especially types 16 and 18 – is involved in the pathogenesis of malignant tumors, with involvement in practically 100% of cervical cancer cases, 85% of anal tumors, and 50% of penile and vulvar tumors.^1^, ^12^ In Brazil, the national HPV vaccination program was instituted in 2014 for girls between 9 and 13 years old, later expanded to boys between 12 and 13 years old.^1^ Vaccination can prevent more than 90% of cases of cervical cancer.^4^

The rates of oral HPV infection in healthy individuals vary between 0% and 20%, depending on the population studied.^15^ HPV is involved in 36% of oropharyngeal squamous cell carcinomas (SCC). When all head and neck carcinomas are included, the prevalence of HPV drops to 26%.^15^

Immunosuppression is an important risk factor for HPV lesions. Studies suggest that temporary changes in the immunity of pregnant women may be the explanation for the higher rates of HPV infection in this group when compared with patients who are not pregnant.^17^ Patients who have received solid organ transplants (SOT) and those who are HIV-positive are especially susceptible. It is estimated that about 40% of transplant recipients will have CW.^6^ The presence of cutaneous HPVs ubiquitously distributed over the skin has been described in this group of patients.^6^ The incidence of skin cancer associated with HPV in these patients is estimated to increase by 5% per year, with a cumulative risk of 44% after nine years.^6^ A similar finding is described regarding the incidence of malignant and premalignant lesions in the mucous membranes, where immunosuppression stands out as an important risk factor.^4^ The risks vary for the various locations of HPV-related neoplasms among patients with SOT and individuals living with HIV, which can reach an incidence of up to 80 times higher than in the general population in the case of anal cancer in some subpopulations.^3^, ^18^, ^19^, ^20^

## Pathogenesis

### Virus characteristics

Over millions of years, PVs have evolved in parallel with the species they have infected, having great cellular specificity for epithelial tissues and an enormous degree of adaptation. They are a group of viruses with marked species-specificity and are capable of producing subclinical infections, of long evolution with low viral replication; clinical infections, through the evasion of the host’s immune mechanisms; in addition, they participate in the process of carcinogenesis of tumors of the cutaneous, anogenital, and oral region.^7^

The Papillomaviridae family includes two subfamilies, Firstpapillomavirinae, with more than 50 genera and 130 species, and Secondpapillomavirinae, with one genus and one species.^21^ The classification is based on the DNA sequence of the L1 gene: genera share more than 60% of the nucleotide sequence and species, between 71% to 89% of the nucleotides. A new type of PV must have its L1 region DNA sequence differentiated by at least 10% from a known type, while a variant has less than 2% difference. Approximately 429 types have been identified, 218 of which are isolated from humans.^2^ The PVs that infect humans belong to five genera: alpha-papillomaviruses (α-HPV), with 14 classified species and 65 types, produce macroscopic visible lesions and comprise viruses of low skin risk and of low and high mucosal risk, the latter being involved in carcinogenesis; beta-papillomaviruses (β-HPV), with six classified species and 52 types, produce subclinical infections, mainly in childhood, and are particularly involved in the onset of skin tumors in immunosuppressed patients with epidermodysplasia verruciformis (EV); gamma-papillomaviruses (γ-HPV), with 27 classified species and 97 types, produce subclinical infections in childhood and clinical lesions in immunocompromised patients; and mu-papillomavirus (μ-HPV), with three species and three types, and nu-papillomavirus (ν-HPV), with one species and one type, are related to skin lesions.^2^, ^21^, ^22^

PV measures approximately 50 nm in diameter and has an non-enveloped capsid with icosahedral symmetry; its DNA consists of a double helix with a genome ranging from 5,748 (*Sparus aurata papillomavirus* type 1; SaPV1) to 8,607 nucleotides (*Canine papillomavirus* type 1; CPV1). The viral capsid consists of 72 pentamers of the main L1 protein and 12 molecules of the secondary L2 protein. It infects fish, reptiles, birds and, mainly, mammals.^21^

PVs are genetically stable. The viral genome is generally divided into an upstream regulatory region (URR) or long control region (LCR) and two groups of open read frames (ORFs) that are designated as early (E) or late (L). The core genes E1, E2, L1, and L2 fulfill essential functions during the life cycle of the virus in the epithelium. The E5, E6, and E7 genes are considered accessory and have evolved to facilitate viral replication in the squamous epithelium, not being present in all types of PVs. The genes encode proteins with specific functions during the viral cycle. The URR or LCR region is located between the ORFs L1 and E6; it contains the origin of viral replication, and is a site for viral and cellular transcription factors. The E1 protein is an ATP-dependent DNA helicase, important in the replication and amplification of the viral genome in the nucleus of infected cells. The E2 protein regulates the viral life cycle, is a co-activator of the viral genome replication by recruiting the E1 protein, and works as a transcription factor for the E6 and E7 proteins. The L1 protein is the main maker of the viral capsid, while the L2 protein, in addition to composing the capsid, is involved in the encapsidation of viral DNA and in the entry and traffic of the virus in the nucleus. The L1 protein is highly immunogenic, and serves as the basis for vaccines used to prevent HPV infection. The E4 viral protein has some characteristics of the core proteins and is expressed in the late stages of the viral cycle; it binds to cytokeratins, producing their rupture and contributing to the release and transmission of the virus. The E5 protein is a hydrophobic transmembrane protein, present in the alpha genus, with a role of evading the immune response and apoptosis. Proteins E6 and E7 are related to the amplification of the viral genome and to carcinogenesis, in some types of HPV.^7^, ^21^ Viral proteins are expressed differently during epithelial differentiation and during the life cycle of the virus, which follows this differentiation (Table 1).

**Table 1:** Human papillomavirus (HPV) genome and viral proteins.

Genome regions	Viral protein	Function
URR^a^/LCR^b^		Origin of viral replication; site for viral and cellular transcription factors
Late ORFs^c^	L1	Constituent of viral capsid
	L2	Constituent of viral capsid; DNA encapsidation and virus entry and transit in the nucleus
Early ORFs^c^	E1	Replication and amplification of the viral genome in the nucleus of infected cells
	E2	Regulates the viral cycle and transcription factor for proteins E6 and E7
	E4	Binds to cytokeratins, contributes to virus release and transmission
	E5	Plays a role in evading the immune response and apoptosis^d^
	E6	Amplification of the viral genome and carcinogenesis^e^
	E7	Amplification of the viral genome and carcinogenesis^e^

a Upstream regulatory region.

b Long control region.

c Open read frames.

d Present in the alpha genus.

e Function in some types of HPV.

### Viral infection and host immune response

HPV infection occurs when viral particles reach exposed basal cells, usually by microtrauma in the epithelium. It shows tropism by stem cells from different mucosal and cutaneous epithelia, whose differences are believed to influence the pattern of gene expression of the virus. The places where the infection appears to be facilitated are: transition epithelium in the uterine cervix and anal region, specialized epithelia of the salivary glands of the oral cavity and tonsillar crypts of the oropharynx, hair follicles, eccrine and apocrine glands, in addition to the epidermis.^7^

The virus binds to the heparan sulfate proteoglycans of the basal cells and exposed basement membrane, which serves as a primary receptor. This binding happens with the L1 protein, which induces changes in the viral capsid that bind it to another receptor, not yet identified. The internalization of the capsid, which occurs similarly to a macropinocytosis mechanism, can take two to four hours. The entry of the viral genome into the nucleus is mediated by the L2 protein. Thereafter, infection begins by viral genome transcription.^22^

The viral replication cycle has three phases: an initial amplification of DNA, with the participation of proteins E1 and E2; a phase of maintenance of viral replication that occurs in the proliferating infected cells; and a phase of genome amplification and formation of new viruses that occurs when the cells complete their differentiation. The maintenance phase can last for months or years. Viral capsid proteins encapsidize viral DNA to form viral particles that are released as epithelial cells are detached.^21^

HPV, in general, completes its life cycle in epithelial cells; does not produce viremia, cell lysis, or inflammation; and remains protected from the immune system through mechanisms of evasion of the innate immune response, in addition to delaying the adaptive immune response. As a rule, this phenomenon is correlated with persistent HPV infections and an increased chance of progression to neoplasms.^7^

Keratinocytes are sentinel cells of the immune system and can initiate a response to viral pathogens. Proteins E6 and E7 from HR-HPV (16, 18, and 31) interfere in this response. HPV infection does not activate or recruit Langerhans cells, antigen-presenting cells present in the squamous epithelium. They do not respond to the HPV capsid, unlike dermal dendritic cells, which are able to initiate an immune response to the viral L1 protein. Consequently, an antigen-specific immune response does not occur.^21^, ^23^

Initial infections of some types of HPV can be controlled by innate immunity, while clinically evident lesions require a local immune response involving CD8+ cytotoxic T lymphocytes and T helper 1 CD4+, which produce interleukin 2 (IL-2) and interferon-gamma (IFN-γ) and recognize the viral proteins E6, E7, and E2. The complete elimination of the virus may not take place, leading to its latency. HPV reactivation is often observed in immunosuppressed transplant patients and HIV patients. In mucosal infections, some other factors have been cited as capable of interfering in the immune response to HPV, such as the microbiota, mucosal secretion products, and hormonal responses.^23^

### Oncogenicity

The first HPVs associated with cancer were HPVs 5 and 8 in patients with EV, a genetic disease that predisposes to infection by HPVs, mainly of the beta genus, and in which, due to a mechanism that is not fully understood, the body fails to eliminate the virus and warts are observed in various areas. In these patients, the HPVs are related to the appearance of non-melanoma skin cancers (NMSC) in areas exposed to the sun. β-HPVs also contribute to NMSC in immunosuppressed SOT patients, and doubts remain about their role in immunocompetent patients.^22^, ^24^ The main NMSC found in these patients was cutaneous SCC, whose main risk factor is ultraviolet radiation (UVR). Studies suggest that β-HPVs act as supporting carcinogens, necessary in the cancer initiation process, since the viral genome is not detected in tumors. They would facilitate the accumulation of DNA mutations induced by UVR.^25^

Studies show that they target cell pathways involved in apoptosis, DNA repair, and cell differentiation. Cellular targets vary between different types of β-HPVs. The E6 protein of HPVs 5, 8, and 38, for example, allows the infected cell to accumulate genetic mutations necessary for the process of carcinogenesis by interfering with cellular defense mechanisms. It binds to the p300 protein, interferes with DNA repair in response to the damage produced by UVR, and contributes to the accumulation of mutations, chromosomal abnormalities, and cellular immortalization. Furthermore, it induces the degradation of Bak, a protein that repairs, through apoptosis, UVR-induced cellular DNA damage. The E7 protein of β-HPVs 23, 38, and 49 also has oncogenic properties, similar to the alpha genus, which involve inhibition of tumor suppressor proteins of the retinoblastoma family.^25^

Alpha genus HPVs, specifically 12 phylogenetically related types of sexual transmission, especially HPV 16, are related to cervical cancer and, with increasing evidence, to other cancers in the anogenital region (anus, vulva, vagina, and penis) and oropharynx. Based mainly on studies on cervical cancer, the following are considered HR-HPVs: HPV 16, HPV 31, HPV 33, HPV 35, HPV 52, and HPV 58, of the alpha-9 species; HPV 18, HPV 39, HPV 45, and HPV 59 of the alpha-7 species; HPV 51, of the alpha-5 species; and HPV 56, of the alpha-6 species.^23^, ^26^ HPVs 16 and 18 are detected in up to 70% of all cervical cancers. There are other factors that contribute to the onset of cervical cancer, such as smoking, multiparity, use of oral contraceptives, and HIV co-infection.^26^ The mechanism by which HR α-HPVs participate in oncogenesis includes a deregulation in viral gene expression, which is caused by the integration of the viral genome in high-grade premalignant lesions and co-expression of proteins E6 and E7. Proteins E6 and E7 modify the cellular environment, allowing the amplification of the viral genome in differentiated epithelial cells, which would normally be incompetent for DNA replication. In most HPVs, the E7 protein binds to the retinoblastoma (pRb) tumor suppressor protein and to the related proteins p107 and p130. In HR-HPV, the E6 protein inactivates the tumor suppressor p53 protein, inhibiting its antiproliferative and apoptotic activities.^22^

This advance in knowledge was fundamental to establish primary and secondary prevention strategies: in 2014, vaccination to prevent HPV infection was introduced in the National Immunization Program of the Brazilian Ministry of Health and molecular tests for HPV DNA research have been introduced in some countries in order to identify high-risk types.^22^, ^26^ The development of preventive measures for tumors related to β-HPVs is still awaited.

### Clinical manifestations

The different clinical manifestations vary according to the type of HPV involved in the infection, the anatomical site of virus predilection, and the host’s immune response. HPV 1 and 63, of the Mu genus, for example, appear to target the eccrine ducts of the palmoplantar regions: HPV 1 causes myrmecia-like warts on the palms and plants, being rare elsewhere; HPV 63 produces punctate warts (0.5 to 2 mm) in the plantar region and is identified in the keratinocytes around the acrosyringia and in the upper portion of the eccrine duct. HPVs 4 and 65, of the gamma genus, produce pigmented warts in the palmoplantar region and lateral region of the hands and feet, while HPV 95, also of the gamma genus, produces non-pigmented papules in the plantar region. HPV 7, of the alpha genus, produces “butcher” warts, mainly on the hands. These considerations also apply to mucosal HPVs. For example, HPVs 6 and 11, belonging to the alpha genus, appear to have a more limited epithelial distribution: HPV 6 has a predilection for the genital region, while HPV 11 is more frequently observed in the oral region. These different epithelial tropisms reflect genetic differences of the virus, in addition to different patterns of gene expression and epithelial regulation in different anatomical sites^7^ (Table 2).

**Table 2:** Clinical manifestations and main types of related HPV.

Clinical manifestation	HPV	References
Cutaneous		
Common wart	2, 4, 7, 26, 27, 28, 29, and 57	Vlahovic, 2016
Butcher’s wart	7	Bacaj, 2018, and Egawa, 2015
Flat wart	3, 10, 27, 28, 29, and 41	Vlahovic, 2016
Palmoplantar warts	1, 2, 4, 7, 63, 65, and 95	Egawa, 2015 and Abeck, 2019
Myrmecia palmoplantar wart	1	Vlahovic, 2016, and Bacaj, 2018
Mosaic palmoplantar wart	2	Vlahovic, 2016, and Bacaj, 2018
Classic epidermodysplasia	5, 8, 3, 9, 10, 12, 14, 15, 17, 19 to 25, 28, 29, 36, 38, 46, 47, 49, and 50	Przybyszewska, 2017, and Huang, 2018
HPV epidermodysplasia associated with malignancy	5, 8, 17, 20, and 47	Przybyszewska, 2017
Non-classical epidermodysplasia (RHOH mutation)	3, 12, and 20	Przybyszewska, 2017, and Huang, 2018
Non-classical epidermodysplasia (MST-1 mutation)	5, 15	Przybyszewska, 2017, and Huang, 2018
Non-classical epidermodysplasia (CORO1A mutation)	5, 17	Przybyszewska, 2017, and Huang, 2018
Non-classical epidermodysplasia (IL-7 mutation)	3	Przybyszewska, 2017, and Huang, 2018
Acquired epidermodysplasia – HIV	5, 8, 14, 19, 20, and 21	Przybyszewska, 2017, and Huang, 2018
Iatrogenic acquired epidermodysplasia	5, 46	Przybyszewska, 2017, and Huang, 2018
β-HPV associated with risk of SCC	5, 8, 15, 17, 20, 24, 36, and 38	Rollison et al., 2019
Immunosuppressed – common warts	2, 27, 57	Martelli-Marzagão, 2016
Mucosa		
Genital wart or condyloma acuminatum	6, 11, 16, 18	Leto, 2011
Bowenoid papulosis	16, 18, 31, 33, 39, and 52	Yoneta, 2000
Oral mucosa – common wart	2, 4	Betz, 2019
Oral mucosa – focal epithelial hyperplasia or Heck's disease	13, 32	Betz, 2019
Oral mucosa – squamous papilloma and condyloma acuminatum	6, 11, 16, and 18	Betz, 2019
Recurrent respiratory papillomatosis	6, 11, 16,18,31,33	Fortes, 2017
Genital warts in children	2	Handley, 1997
Giant condyloma acuminatum	6, 11, 16,18,33	Spinu, 2014
Oral florid papillomatosis	2, 3, 6, 11	Pãtrascu, 2016
Immunosuppressed – genital warts	6, 11, and 16	Brazil, 2018 page 11; Burd, 2016

### Clinical manifestations on the skin

The clinical manifestations on the skin include those common in medical practice, but there are also rarer manifestations, many of them related to genetic defects, which have an impact on the host's response to the virus infection. At the same time, the special situation of immunosuppression (whether acquired or not) is noteworthy, interfering with the natural history of the infection, leading to extensive conditions that are refractory to treatment and amplifying the oncogenicity of HPV.

## Benign skin lesions

### Cutaneous warts

CWs are a benign condition and present spontaneous resolution in most cases. Nonetheless, they can grow, cause discomfort or embarrassment to patients, and persist for months or years, increasing viral transmission between individuals. The denomination of the warts, in general, takes into account the appearance of the lesion, such as flat and filiform wart; the location, such as palmoplantar, periungual, or genital; or the group of individuals in which the wart is mostly found, such as butcher’s wart. In relation to CWs, in general, three entities are considered: common wart, flat wart, and palmoplantar wart.^27^, ^28^

### Common wart

**Figure 1:**
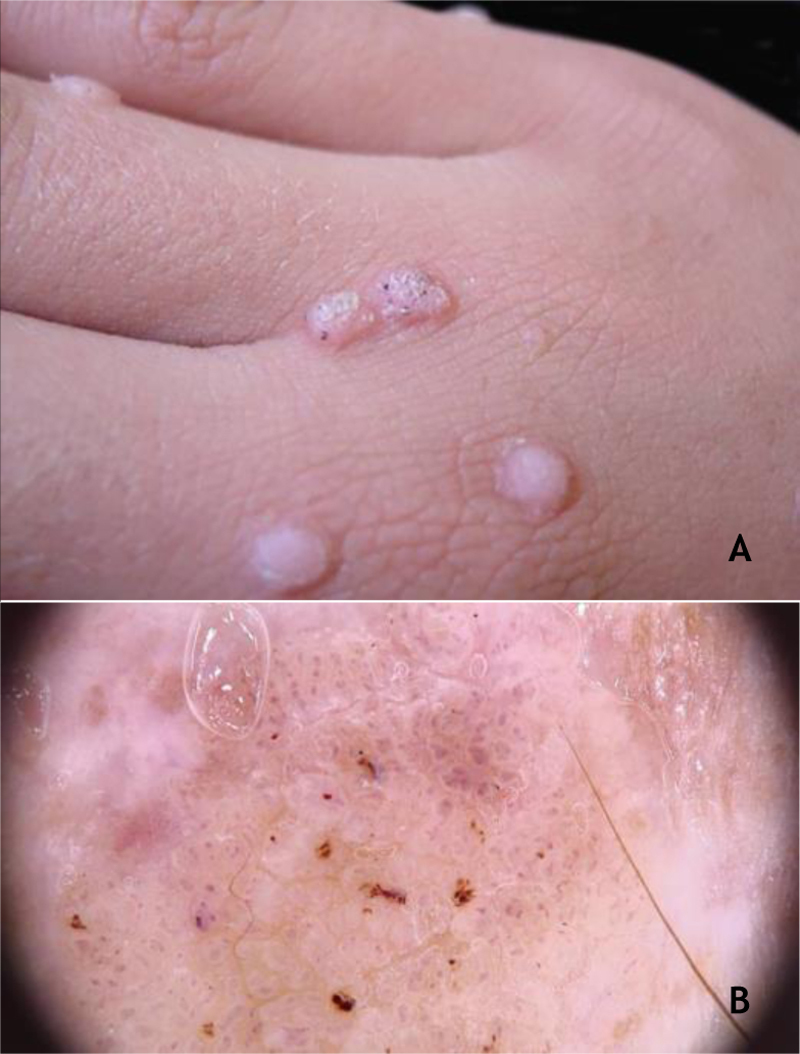
(A), Common wart, papules with keratotic surface, some with dark spots. (B), common wart at dermoscopy, vessels surrounded by a white halo and hemorrhagic dots. Source: Dermatology Service of HC-UFMG/EBSERH.

Clinically, common warts (CmWs) present as papules or raised nodules, with a rough surface, skin-colored or yellowish, single or multiple, of varying sizes, which can converge and form large plaques (Fig. 1A). A typical finding is the presence of black dots on the surface of the lesion that correspond to epidermal hemorrhage or dilated capillary loops inside elongated dermal papillae.^28^, ^29^ They can occur anywhere in the integument. They are most common in exposed areas, such as the back of hands, fingers, knees, and elbows. They are usually asymptomatic and have an unpredictable evolution, and may remain unchanged for months or years, develop a large number of new lesions in a short period of time, or undergo spontaneous regression.^27^, ^28^ The filiform wart is a morphological variant distinct from the CmW that is most commonly observed on the beard, nose, and periocular region, as filiform keratotic projections, with perpendicular or oblique growth on the skin surface. They can be isolated or multiple.^28^, ^29^ The butcher's wart is a rare CmW associated with HPV 7, which receives this name because it is found in butchers or meat and fish handlers, whose clinical presentation is the cauliflower appearance. It occurs mainly in the hands of these professionals. Maceration and trauma appear to be the predisposing factors.^7^, ^29^

The HPVs most commonly found in CmWs are HPV 2, 4, 7, 26, 27, 28, 29, and 57.^8^ Dermoscopy shows papillae grouped with punctate or looping vessels and/or dots and hemorrhagic lines, often surrounded by a white halo. Filiform warts show similar dermoscopy, with a predominance of papillae^30^ (Fig. 1B). CmWs must be differentiated from seborrheic keratosis, lichen planus, nodular prurigo, and also from neoplastic lesions such as SCC.^28^, ^29^

### Flat wart

Flat warts appear as flat, skin-colored, or yellowish erythematous papules, with a flat and smooth surface; they measure a few millimeters and have a tendency to coalesce. Koebner's phenomenon is observed relatively frequently. They usually occur on the face and back of the hands, and are found mainly in children and adolescents (hence the name juvenile flat wart), as well as in immunocompromised patients, such as those living with HIV and SOTs.^28^, ^29^ The HPVs most frequently found are HPV 3, 10, 27, 28, 29, and 41.^8^ Dermoscopic findings include small punctate vessels, distributed on a background without structures, of yellowish color.^30^ Differential diagnoses include flat seborrheic keratosis on the face and molluscum contagiosum, which can be distinguished by its central umbilication.^27^, ^28^

### Palmoplantar wart

Palmoplantar warts (PPWs) appear as hyperkeratotic papules that, on pressure areas, show endophytic growth and, frequently, hemorrhage points. The lesions can be deep or superficial and, when located on the pressure areas, are very painful. They affect children and adolescents more frequently.^27^, ^28^, ^29^ In plantar warts, the infection results from direct contact with HPV, but some authors consider that it can occur through indirect sources, such as walking barefoot in swimming pools or shared bathrooms, or sharing socks, shoes, towels, and sports equipment. Hyperhidrosis of the feet is associated with an increased risk of acquiring PPW.^31^

The myrmecia wart is a deeper lesion that presents a rough surface, surrounded by a hyperkeratotic ring. They are compared to an iceberg, where only a small part is clinically visible, while most of the lesion is found in the deeper layers. They can be single or multiple, with an inflammatory and painful presentation. Myrmecia PPW is associated with HPV 1.^8^, ^29^

**Figure 2:**
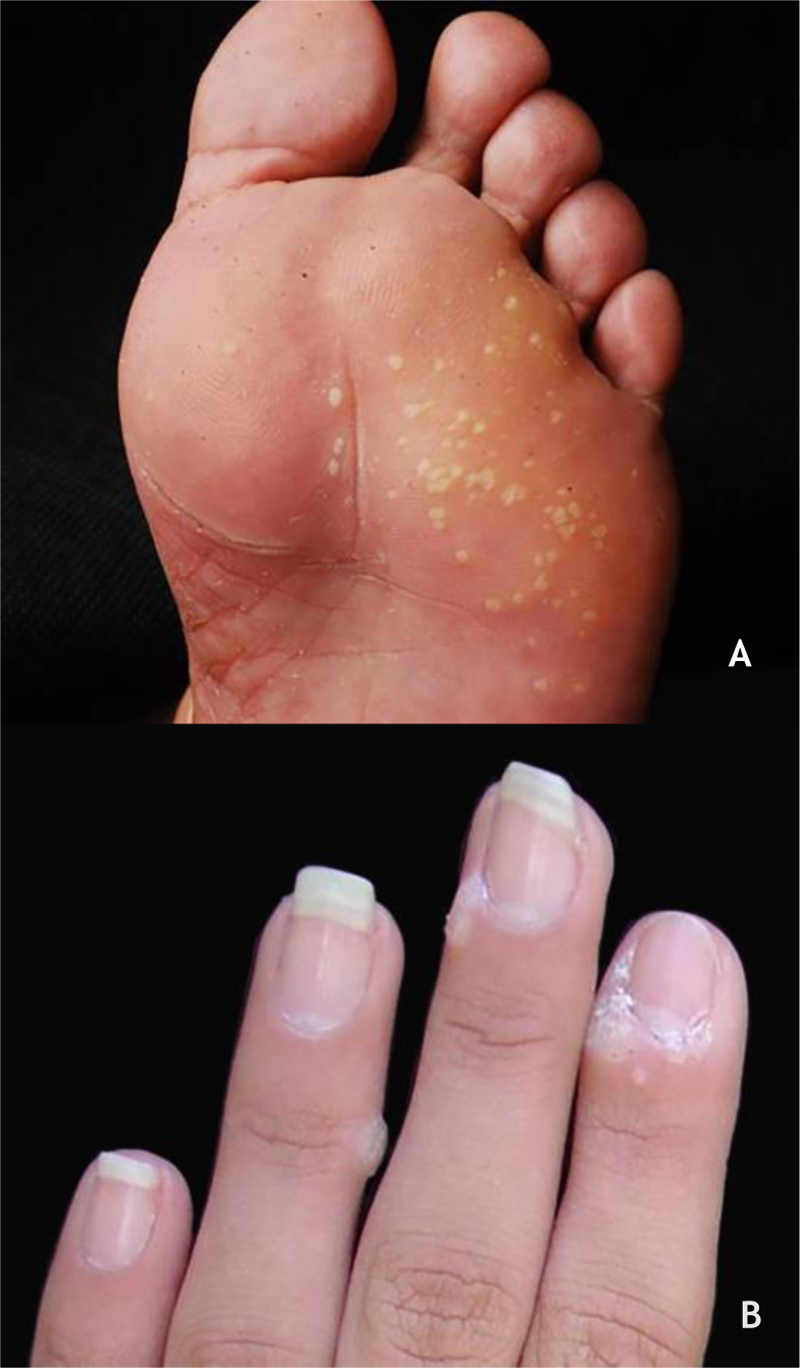
(A), Mosaic plantar wart. (B), periungual wart. Source: Dermatology Service of HC-UFMG/EBSERH.

Mosaic warts are multiple lesions that converge and form keratotic plaques. They are present on the plantar region, with a more superficial and painless presentation, and are caused by HPV 2^8^, ^29^ (Fig. 2A).

Periungual warts, which are usually painful, may show subungual growth resulting in partial onycholysis^28^ (Fig. 2B).

**Figure 3:**
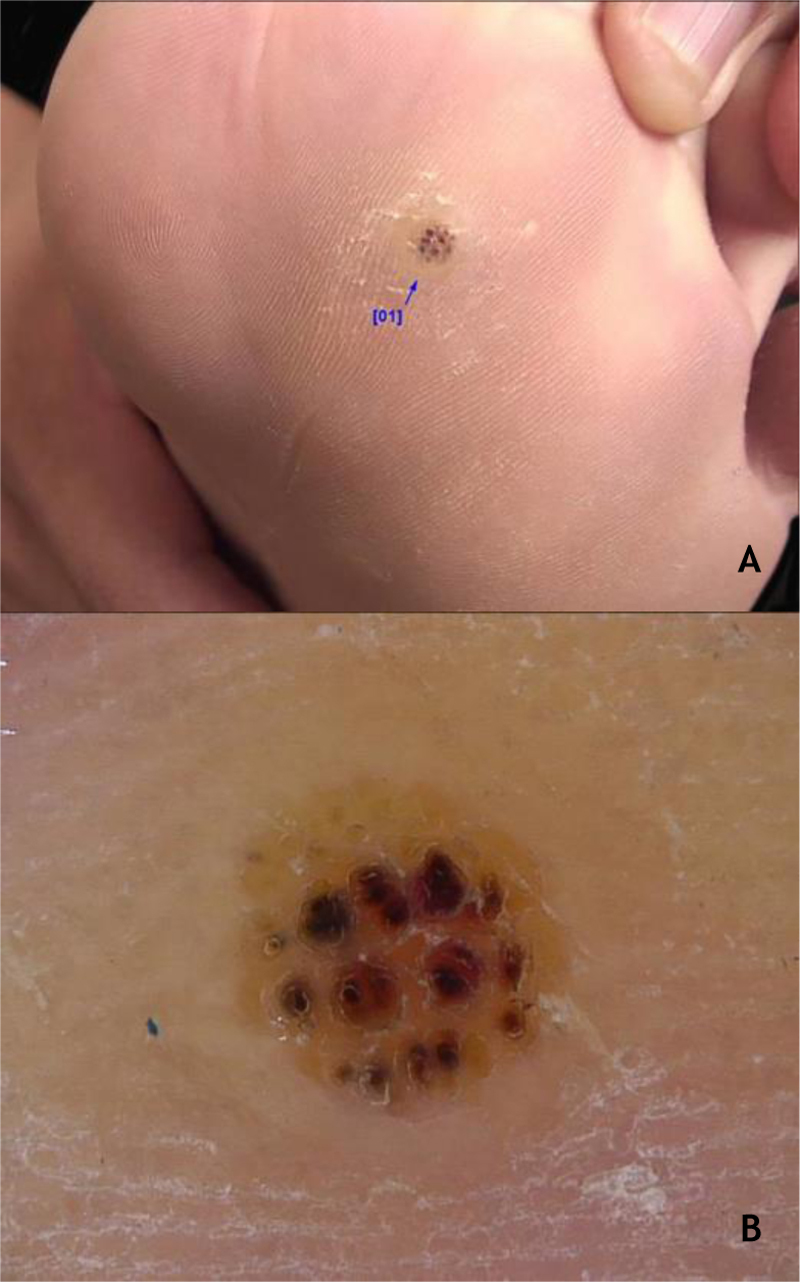
(A), Myrmecia plantar wart (arrow). (B), Plantar wart at dermoscopy, hemorrhagic dots on a yellowish background with interruption of the skin lines. Source: Dermatology Service of HC-UFMG/EBSERH.

The HPVS most commonly found in PPW lesions are types 1, 2, 4, 7, 63, 65, and 95.^7^, ^28^ Dermoscopy in PPWs shows a yellowish or white background with no structures, and small hemorraghic dots that correspond to thrombosed vessels with interruption of the skin lines^30^ (Fig. 3A and B).

Regarding the differential diagnoses, the following should be considered: grooved and punctate plantar pitted keratolysis, punctate palmoplantar keratoderma, calluses, and SCC, especially the verrucous carcinoma subtype.^8^ The identification of thrombosed capillaries and hemorrhagic dots in the wart help to differentiate it from plantar calluses, which do not contain these elements. In addition, a key feature of PPWs is the breakdown of dermatoglyphs at the wart site, which are restored with the resolution of the lesion.^8^ In relation to the periungual wart, the following differential diagnoses are possible: paronychia, digital mucous cyst, subungual keratoacanthoma, and SCC.^28^, ^29^

### Epidermodysplasia verruciformis (EV) and generalized verrucosity

**Figure 4:**
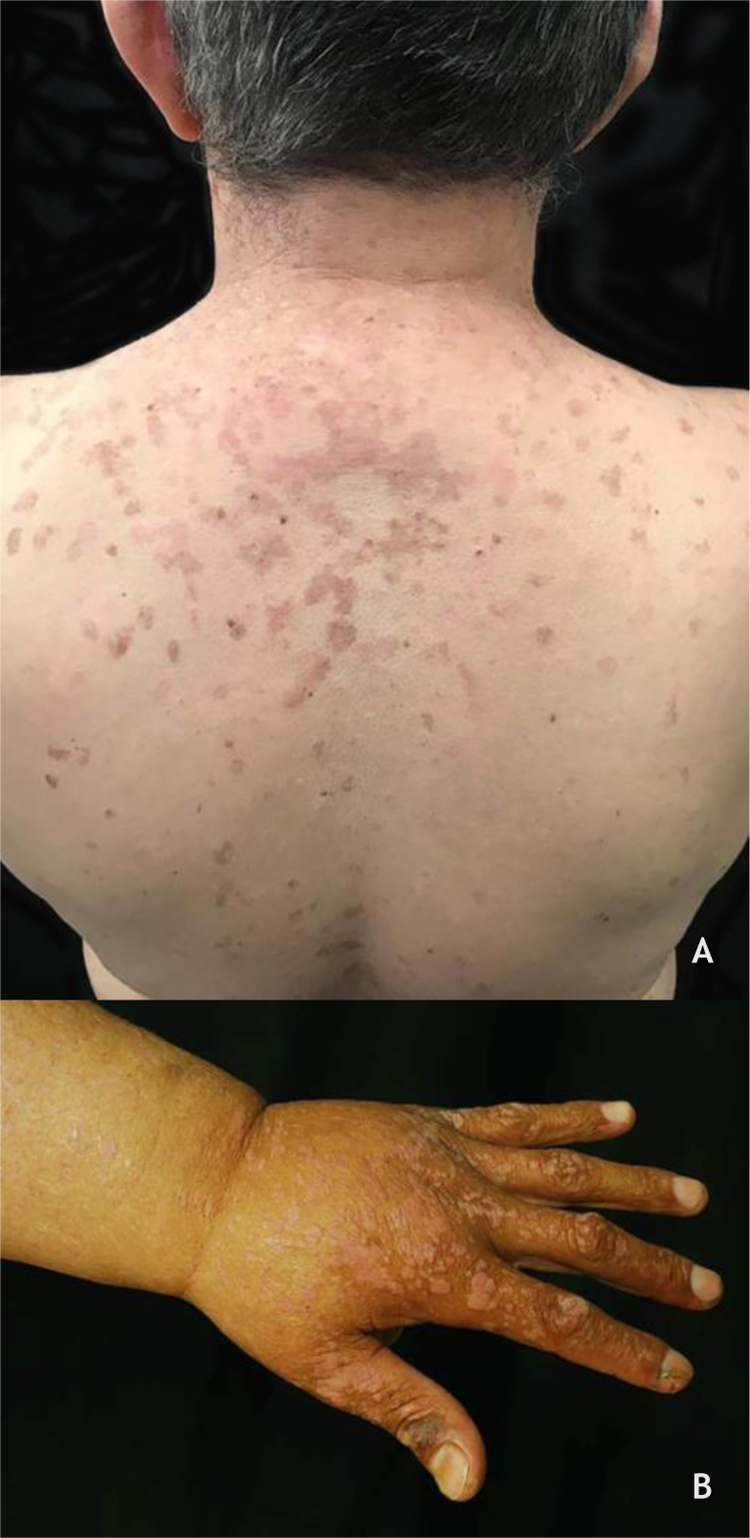
(A), Epidermodysplasia verruciformis, erythematous and/or brownish plaques that resemble pityriasis versicolor lesions and seborrheic keratosis on the trunk. (B), WILD syndrome, numerous flattened erythematous papules forming plaques on the back of the hand and forearm, associated with lymphedema of the upper limb. Source: Dermatology Service of HC-UFMG/EBSERH.

EV, described by Lewandoswsky and Lutz in 1922, is an autosomal recessive genetic disease characterized by a mutation in the TMC6 (EVER1) and TMC8 (EVER2) genes, which leads to chronic infection by specific types of HPVs, mainly of the beta genus.^32^ In EV of the classic form, in addition to HPV 5 and HPV 8, HPVs 3, 9, 10, 12, 14, 15, 17, 19 to 25, 28, 29, 36, 38, 46, 47, 49, and 50 are observed, as well as the Merkel cell polyomavirus.^32^, ^33^ The TMC6 and TMC8 genes encode proteins that inhibit fundamental transcription factors for HPV gene expression.^32^ The classic clinical picture begins in childhood or early adolescence, manifesting itself as erythematous papules or plaques similar to flat warts on the extremities and erythematous and/or brownish plaques that resemble lesions of pityriasis versicolor and seborrheic keratosis on the trunk, neck, and face. The lesions tend to spread throughout the body (Fig. 4A). The disease is associated with an increased risk of the onset of NMSC, especially SCC, in about 30% to 70% of patients, usually from the fourth decade of life onwards, in areas exposed to the sun.^32^, ^33^ HPVs associated with malignant transformation are types 5, 8, 17, 20, and 47.^32^ The main differential diagnoses are: flat warts, pityriasis versicolor, confluent and reticulated papillomatosis (Gougerot-Carteaud syndrome), disseminated superficial porokeratosis, and Darier's disease.

Recently, new mutations have been described in patients with clinical features similar to those of EV, in which the previously reported mutations (RHOH, MST-1, CORO1A, and IL-7) had been ruled out. All of these patients have deficiencies in cellular immunity against HPV infections in common. Patients with these new mutations are also predisposed to other infections and tumors.^32^ In 2018, Huang et al. proposed the following classification: classical genetic EV, in which there is a mutation of the TMC6 and TMC8 genes; non-classical genetic EV, when mutation is observed in other genes, such as RHOH, MST-1, CORO1A; and acquired EV.^33^ In acquired EV, the family history of the disease is missing and a defined secondary cause is found, for example, immunodeficiency due to HIV infection, or iatrogenic, caused by immunosuppressive drugs used in chemotherapy, transplant patients, graft *vs.* host disease, atopic dermatitis, and systemic lupus erythematosus.^33^ In the non-classical form, in the RHOH mutation are observed HPV types 3, 12, and 20; in the MST-1 mutation, types 5 and 15; in the CORO1A mutation, types 5 and 17; and in the IL-7 mutation, type 3. In the acquired form, HPVs 5, 8, 14, 19, 20, and 21 are found in association with HIV; and in the iatrogenic form, types 5 and 46^32^, ^33^ (Table 3).

**Table 3:** Epidermodysplasia verruciformis: classification and related HPV types.

Type	Mutation	Types of HPV
Classical genetic EV	TMC6 (EVER1) and TMC8 (EVER2)	5, 8, 3, 9, 10, 12, 14, 15, 17, 19 to 25, 28, 29, 36, 38, 46, 47, 49, and 50
Non-classical genetic EV	RHOH	3, 12, and 20
	MST-1	5 and 15
	CORO1-A	5 and 17
	IL-7	3
Type	Secondary immunodeficiency	Types of HPV
Acquired EV	HIV	5, 8, 14, 19, 20, 21
	Drug-related	5 and 46

Generalized verrucosity (GENV) was recently described as a chronic and progressive skin infection by HPV, in which patients present over 20 lesions located in more than one region of the body, with impairment of fingers and limited function. The clinical manifestations depend on the type of HPV involved, ranging from lesions such as flat wart, CmW, periungual wart, or genital wart, and are commonly found in several immunodeficiency syndromes. In WILD syndrome, the patient presents warts, immunodeficiency, lymphedema, and anogenital dysplasia (Fig. 4B); in WHIM syndrome, in addition to warts, hypogammaglobulinemia, infections, and myelokathexis are found. GATA2 deficiency, an autosomal dominant immunodeficiency, is associated with monocytopenia, disseminated mycobacteriosis, and opportunistic fungal and viral infections, including HPV. The syndrome caused by DOCK8 deficiency, idiopathic CD4 lymphocytopenia, and the syndromes previously described are considered types of GENV. GENV differs from EV by clinical and histopathological parameters, and the HPV types involved.^32^, ^34^

## Malignant skin lesions

### Verrucous carcinoma of the skin

**Figure 5:**
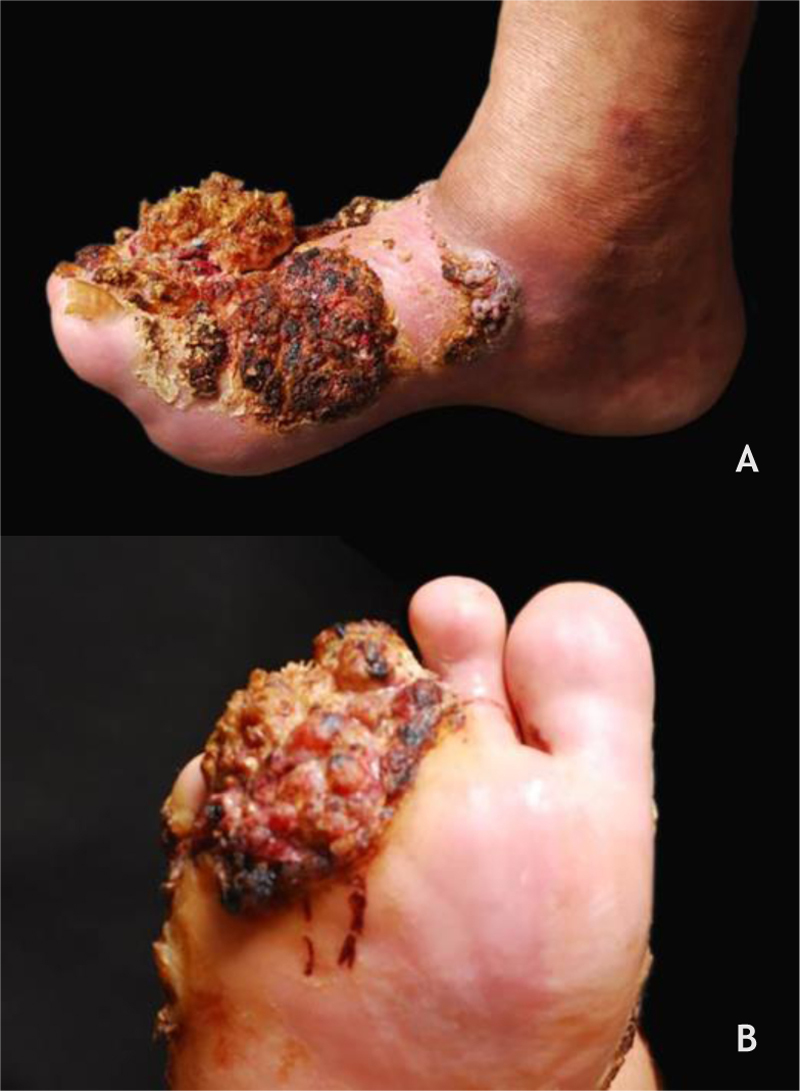
(A and B) Verrucous carcinoma, large tumor in the forefoot, multilobulated, with verrucous areas, reaching the dorsum of the foot (A) and sole (B). Source: Dermatology Service of HC-UFMG/EBSERH.

Verrucous carcinoma, described by Ackerman in 1948, is considered a variant of SCC with insidious evolution, slow-growing but recurring. The cutaneous form, epithelioma cuniculatum, is most commonly observed on the feet, predominates in men (2.3:1), and has a mean age of lesion onset of roughly 55 years. Its clinical presentation can vary from a small keratotic papule, similar to a common wart, to large, multilobulated tumors with a cauliflower aspect; differential diagnosis should include plantar warts, keratinocytic tumors such as keratoacanthoma, basal cell and squamous cell carcinoma, subcutaneous mycoses, and others (Fig. 5A and B). HPV is a potential contributing factor for tumor onset, and the association with types 2 and 16 has been described. The metastatic potential is low and extensive excision of the lesion or micrographic surgery is recommended.^35^

### Clinical manifestations on the mucous membranes

Most of the time, HPV infections are subclinical.^36^ Approximately 1% to 2% of the population has anogenital warts and 2% to 5% of women show changes in the Pap smear caused by HPV infection.^37^ Lesions caused by low-oncogenic-risk HPVs (LR-HPV) are generally associated with low-grade squamous intraepithelial lesions (LSILs), equivalent to the histopathological picture of mild dysplasia or cervical intraepithelial neoplasia grade 1 (CIN 1). HR-HPVs are generally associated with high-grade squamous intraepithelial lesions (HSILs), corresponding to moderate dysplasia (CIN 2) and severe dysplasia or carcinoma *in situ* (CIN 3). Other epithelia may suffer the oncogenic action of the virus, originating vaginal intraepithelial neoplasia (VAIN), penile intraepithelial neoplasia (PIN), anal (AIN), and vulvar (VIN), currently called vulvar HSILs when related to HPVs and NIVd – differentiated, when linked to other precursor lesions. Flat lesions with basal atypia and koilocytosis due to HPV are called LSILs.^37^, ^38^

### Benign lesions in the anogenital mucosa

**Figure 6:**
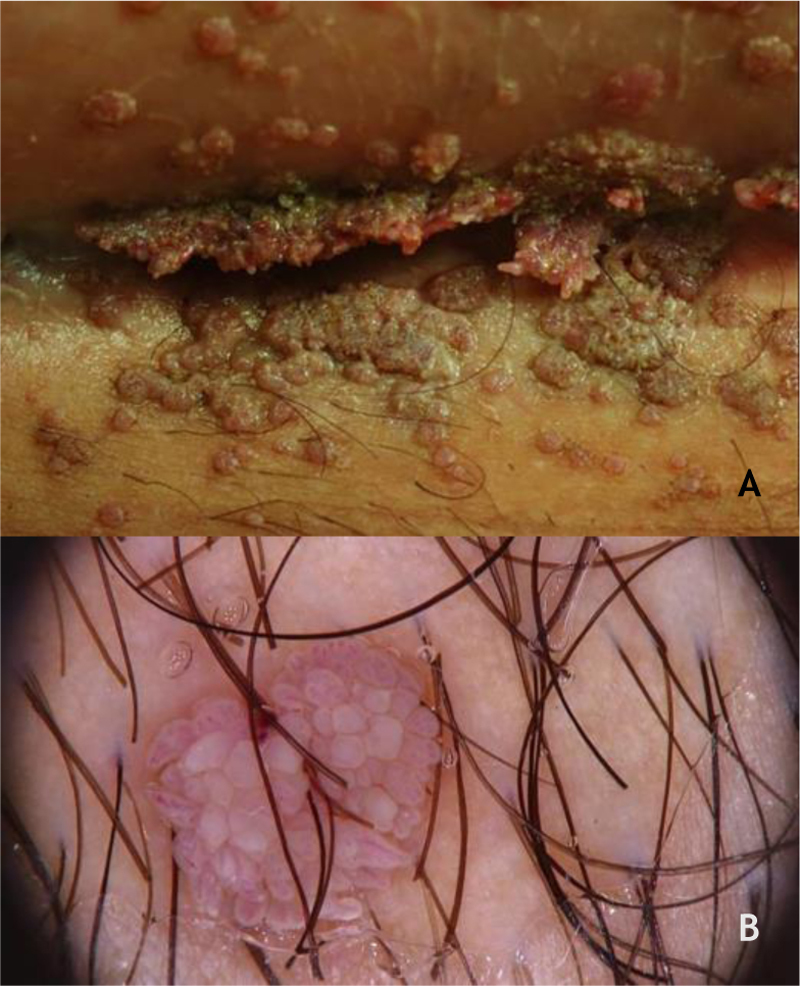
**(**A), Condyloma acuminatum, papules and plaques, vegetating, skin-colored, pinkish and brownish, located in the suprapubic fold. (B), Condyloma acuminatum at dermoscopy, vessels surrounded by a white halo in digitiform projections. Source: Dermatology Service of HC-UFMG/EBSERH.

Genital warts (GW) or condyloma acuminatum are the most common clinical manifestation of HPV infections in the anogenital region. They are related to LR-HPVs 6 and 11, although co-infection with HR-HPV (16, 18, and others) are described.^39^ Their size varies from millimeters to several centimeters; therefore, from papules to plaques, often with vegetation leading to the aspect described as cauliflower. They can be skin colored, pinkish, or brownish. In addition to the anogenital region, they can also be located in inguinal and suprapubic folds, as well as in the oral cavity by orogenital transmission. Symptoms such as pain, itching, or bleeding are unusual.^36^, ^37^ Most of the time, they can be diagnosed with the naked eye, but they must differentiated from several dermatoses such as lichen planus, seborrheic keratosis, molluscum contagiosum, pearly penile papules, achrocordons, and syphilis flat condyloma. Tumor lesions should also be considered as a differential diagnosis.^38^ Dermoscopy can be useful: the most common pattern is mosaic, with the presence of glomerular vessels or dots grouped in the center, surrounded by a whitish network. Elevated lesions may present a digitiform aspect (Fig. 6A and B). On dermoscopy, pigmented GW may have a cerebriform pattern or resemble seborrheic keratosis.^30^ In some cases, biopsy may be necessary.

**Figure 7:**
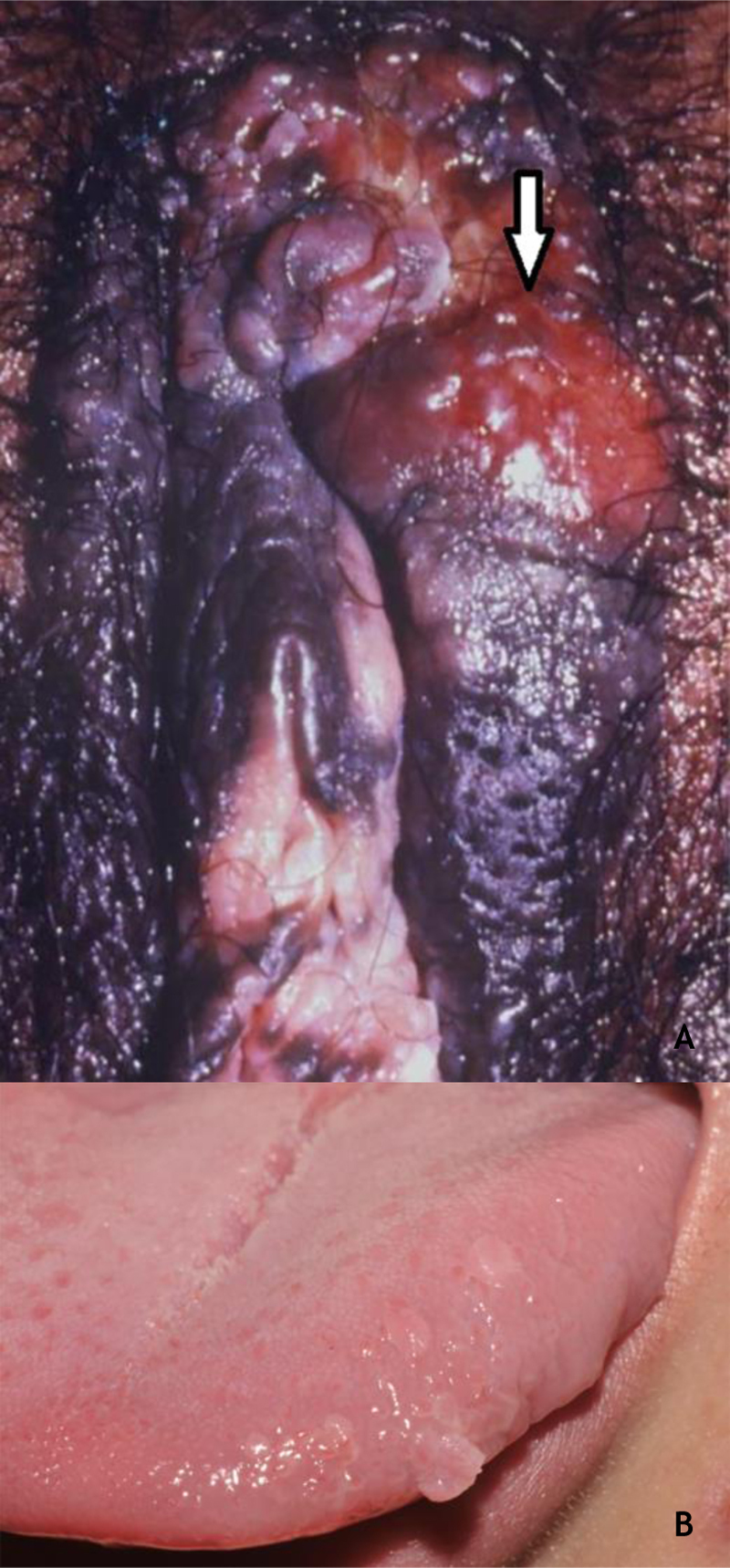
(A), Bowenoid papulosis, multiple hyperpigmented plaques on the female genitalia in an immunocompetent patient, with an infiltrative and eroded tumor (arrow). Source:Dermatology Service of HC-UFMG/EBSERH. (B), Oral condylomas, flattened papules, and pedunculated lesion on the lateral border of the tongue. Source: Dermatology Discipline, Botucatu Medical School - UNESP.

Bowenoid papulosis (BP) occurs in both sexes, preferentially affecting young people who are sexually active. Depending on its location and histopathological picture, it may correspond to LSILs or HSILs of the vulva and PIN or AIN. Histopathologically, it resembles SCC *in situ*, as described in part II of this review. Clinically, it is characterized by flat, hyperchromic or erythematous, multiple, asymptomatic papules, located in the anogenital region, resembling seborrheic keratosis, common wart, lichen planus, or melanocytic nevus.^39^ BP dermoscopy reveals a pattern without structures, with dotted or glomerular vessels, arranged in lines. This pattern is often combined with white, brown, or gray structureless areas. Scales may be observed.^27^ Although HPV 16 is the most implicated type, other HPVs, such as 18, 31, 33, 39 and 52, have also been reported.^40^ It has a benign course, often with spontaneous regression, especially in young males. It may be more aggressive in elderly and immunocompromised patients (Fig. 7A). For therapeutic purposes, it should be kept in mind that, despite many atypias on histopathology, the evolution is favorable in most cases.^39^, ^40^

### Benign lesions on the oral mucosa

As in other locations, most HPV infections in the oral cavity are subclinical. The incidence of clinically defined lesions is estimated at around 3%. Lesions of the oral mucosa caused by HPV include CmW, condyloma acuminatum, squamous papilloma (SP), and focal epithelial hyperplasia (FEH). These lesions have many clinical and histopathological characteristics in common. HPVs 2 and 4 are considered to be related to CmWs of the oral mucosa, while in FEH, HPVs 13 and 32 were implicated; types 6 and 11 are common in SP and condyloma acuminatum. In the latter, HPVs 16 and 18 have also been described.^36^ The characterization of SP and condyloma acuminatum as distinct clinical entities remains controversial, considering the clinical presentation and types of HPV^36^ (Fig. 7B). CmW is rare in the oral mucosa and is thought to occur by autoinoculation, especially in children; it is preferentially located on the lips and palate, usually single, pink or whitish, and exophytic. SP predominates in adults, has the palate and tongue as preferred locations, and is typically an exophytic lesion, with a digitiform or cauliflower aspect and up to 5 mm in size. The differential diagnosis includes CmW, condyloma acuminatum, verruciform xanthoma, and SCC, among others. Thus, biopsy for histopathology of these lesions is mandatory. Condyloma acuminatum in the oral mucosa is more rare, and may or may not be associated with anogenital lesions. The preferred transmission is orogenital, but it can occur through contact with objects. Usually, multiple lesions, sessile, whitish or pink, in the upper lip or tongue are observed.^36^ Anamnesis and a complete physical examination, including the genital and perianal regions, are essential, although biopsy may be necessary.

FEH, or Heck's disease, is an uncommon asymptomatic condition that affects the mouth mucosa and predominates in children and young individuals. It was first described in Native Americans and Eskimos, but it exists in several other populations, including the elderly and AIDS patients. Clinically, multiple lesions are described, with predominance of papules, pink or white, flat and sessile; they can also be papillomatous, with a prominent rough surface. The latter are more rare and are located on the tongue and gums, while the former predominate on the labial and jugal mucosa. The lesions are asymptomatic and tend to spontaneous regression. Its manifestation in children should alert for the differential diagnosis with some syndromes such as type 1 neurofibromatosis, Cowden’s syndrome, and type 2B multiple endocrine neoplasia.^36^, ^39^

### Recurrent respiratory papillomatosis

Recurrent respiratory papillomatosis (RRP) is a rare but potentially life-threatening disease that affects the respiratory tract, with a predilection for the larynx and trachea. The juvenile form manifests itself until the age of 20, and its diagnosis can be challenging since it shares symptoms with several common childhood respiratory diseases. It is attributed to infection by HPVs 6 and 11 in more than 90% of cases and, in childhood, it is assumed that the contamination for most cases occurs through the birth canal, with the possibility of infection through the placenta. Other HR-HPVs such as 16, 18, 31, and 33 may also be involved. It has an unpredictable course, and in rare cases evolve with malignancy. The diagnosis is made by histopathology of lesions in the bronchial tree sampled at bronchoscopy. In RRP forms with diffuse involvement of the bronchial tree, the differential diagnoses include Wegener's granulomatosis, recurrent polychondritis, amyloidosis, neurofibromatosis, tuberculosis, and sarcoidosis.^41^

## Malignant mucosal lesions

### Carcinomas in situ

While the terminology is not consensual, Bowen’s disease (BD) and erythroplasia of Queyrat (EQ) correspond to SCCs *in situ* and can be associated with HR-HPVs, especially 16. BD is characterized by erythematous and desquamative plaques located in the keratinized areas of the anogenital region. EQ appears as shiny, erythematous plaques with a velvety appearance in areas of the mucosa of the vulva, glans, and internal aspect of the foreskin. Both can progress to invasive SCC.^39^

### Verrucous carcinomas of the mucosa

#### Buschke-Löwenstein tumor (BLT) and florid oral papillomatosis (FOP)

Giant condyloma acuminatum, also called BLT, is a rare form of anogenital condyloma related to HPV, especially types 6 and 11, but occasionally HR-HPV can be found (16, 18, and 33).^42^ Along with FOP, they comprise verrucous mucosal carcinomas.^42^, ^43^

**Figure 8:**
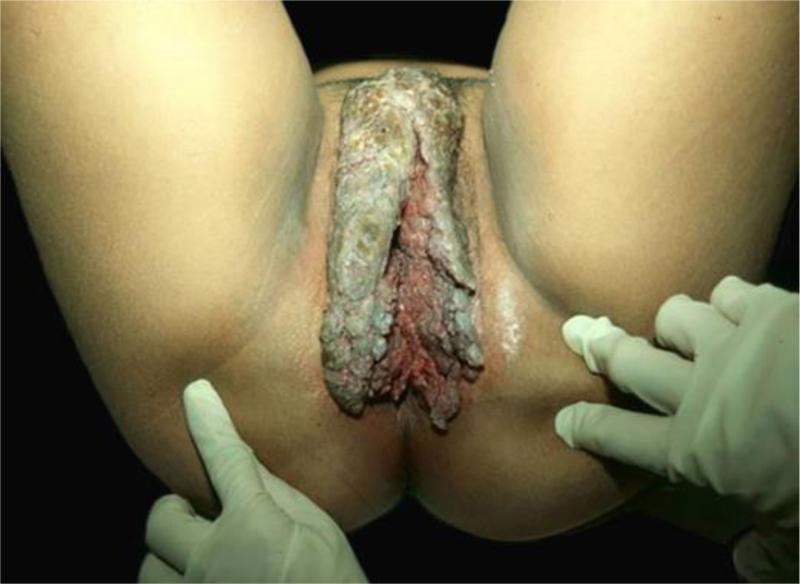
Giant condyloma acuminatum, extensive tumor with vegetating and verrucous areas. Personal Archives: Dra. Ana Tereza Orsi.

The classic description of BLT is a vegetative or verrucous tumor lesion, with exophytic and endophytic growth, sometimes with fistulas and bleeding (Fig. 8). It grows slowly; however, despite its benign histopathological characteristics, it can be aggressive locally and generate metastases.^42^, ^43^ Recurrences are frequent. It predominates in men; its occurrence in children emphasizes the possibility of vertical or peripartum transmission. The incidence of BLT has been increasing and is associated with states of immunosuppression, particularly HIV/AIDS. Other risk factors are smoking, multiple sexual partners, anaerobic infections, chronic local inflammation, presence of phimosis, and other immunodeficiencies.^4^, ^42^, ^43^

The diagnosis is confirmed by the typical histopathology, and must be complemented with imaging exams to define the treatment. FOP occurs preferentially on the cheeks, floor of the mouth, gums, and more rarely on the tongue, palate, and labial commissures. It is more common in men from the seventh decade of life onwards. The use of alcohol and tobacco, the latter both in the form of cigarettes and chewing tobacco, poor oral hygiene, repeated trauma due to inadequate prostheses, in addition to chronic inflammatory diseases such as lichen planus and lupus erythematosus, have been identified as risk factors. The related HPVs are types 2, 3, 6, and 11. Recurrences are frequent during follow-up of patients and, rarely, local metastases occur.^44^

## Clinical manifestations in special situations

### HPV in pregnancy

It is assumed that the hormonal changes that occur during pregnancy temporarily interfere with the immune response, which could impact HPV replication/clearance. Thus, it is expected that there will be an increase in the frequency and progression of genital lesions caused by HPV during the gestational period.^17^, ^37^ In a study conducted in Brazil that compared pregnant and non-pregnant women regarding the presence of HPV in cervical material, Salcedo et al. found a significant difference between the groups studied, showing greater positivity for HPV among pregnant women (25.3% *vs*. 13%). Although no cases of HSILs or carcinoma were found, the authors emphasized the need to monitor HPV infections in this group.^17^

In relation to GWs, typical condylomatous lesions can be normochromatic, erythematous, or brownish with a rough surface, and can be flat, papulous, or pedunculated. They occur most frequently around the vaginal introitus, but can occur in multiple locations, such as the cervix, urethra, perineum, or intrarectal region. GWs can proliferate and become friable during pregnancy. In these patients, the lesions may be larger, more numerous, resistant to treatment, or recurrent.^37^ Its management must be individualized and will be discussed in the treatment section. In pregnant women with condyloma acuminatum, the mode of delivery depends on the obstetric condition. In Brazil, the guideline is that the abdominal route would only be indicated in cases of massive lesions that cause obstruction to childbirth or put the mother at risk of major bleeding.^37^

### HPV in children

The incidence of anogenital warts in children has increased in recent decades.^45^ While in adults and adolescents transmission occurs almost exclusively through sexual contact, it is admitted that in children there are three modes of transmission: perinatal (intrauterine and during childbirth), horizontal (self- and hetero-inoculation, known as “innocent”), and sexual abuse. Therefore, the diagnosis of anogenital warts in children requires an accurate investigation to determine the mode of contamination and to rule out the possibility of sexual abuse. Transmission by fomites does not appear to be relevant.^45^, ^46^

The treatment of children with GW is a challenge in clinical practice, considering the possibility of sexual abuse, whose probability increases directly with increasing age. A retrospective study showed that the positive predictive value relating the presence of HPV infection in the anogenital region and possible sexual abuse increases from 36% between 4 and 8 years to 70% after 8 years of age.^47^ The risk for sexual abuse is higher in girls than in boys, at a 3:1.7 ratio.^45^

GWs in infants and children usually appear in the perineal region and, less frequently, in the vagina, vulva, and penis. Studies indicate that HPV 2, the causal agent of hand warts in humans, is commonly associated with anogenital warts in children, a finding that suggests autoinoculation and heteroinoculation as common ways of acquiring anogenital warts.^48^

Nonetheless, the presence of GWs in a child should alert the caregiver/health professional to the possibility of sexual abuse.^37^ HPV typing does not determine how the virus is transmitted and cannot be used to determine whether or not sexual abuse is present. Any viral type can be transmitted through abuse, innocent contact, or vertical transmission.^45^ History-taking, careful assessment of the socio-clinical context, physical examination, and interdisciplinary teamwork remain the best way to identify possible sexual abuse.^45^ The presence of lesions in family members is not evidence of abuse, and the absence of lesions in a potential abuser does not rule out this possibility. The management of these cases must be individualized and judicious.^37^

The differential diagnoses of condyloma acuminatum in children include condyloma lata, molluscum contagiosum, achrocordon, pseudowarts, or infantile gluteal granuloma. Spontaneous resolution of GWs occurs in most cases in a few years or months; therefore, the therapeutic approach in childhood should be more conservative and less aggressive.^45^

### HPV in the immunosuppressed

Immunosuppressed individuals, especially those with HIV co-infection or immunosuppression due to SOT, have experienced better quality and longer life expectancy. The persistence of HPV infection is greater in these individuals than in the general population, which, combined with immunosuppression itself and the higher frequency of HR-HPV, may result in a higher incidence of neoplasms.^18^, ^19^

Common and benign diseases associated with HPV, such as GWs and extragenital warts, are also more prevalent in this population than in the general population.^4^, ^18^, ^19^, ^32^ The failure of the immune system to process and adequately react to HPV infection in immunocompromised individuals can result in extensive, severe, and persistent manifestations of the diseases related to the virus.^4^

### Cutaneous warts

**Figure 9:**
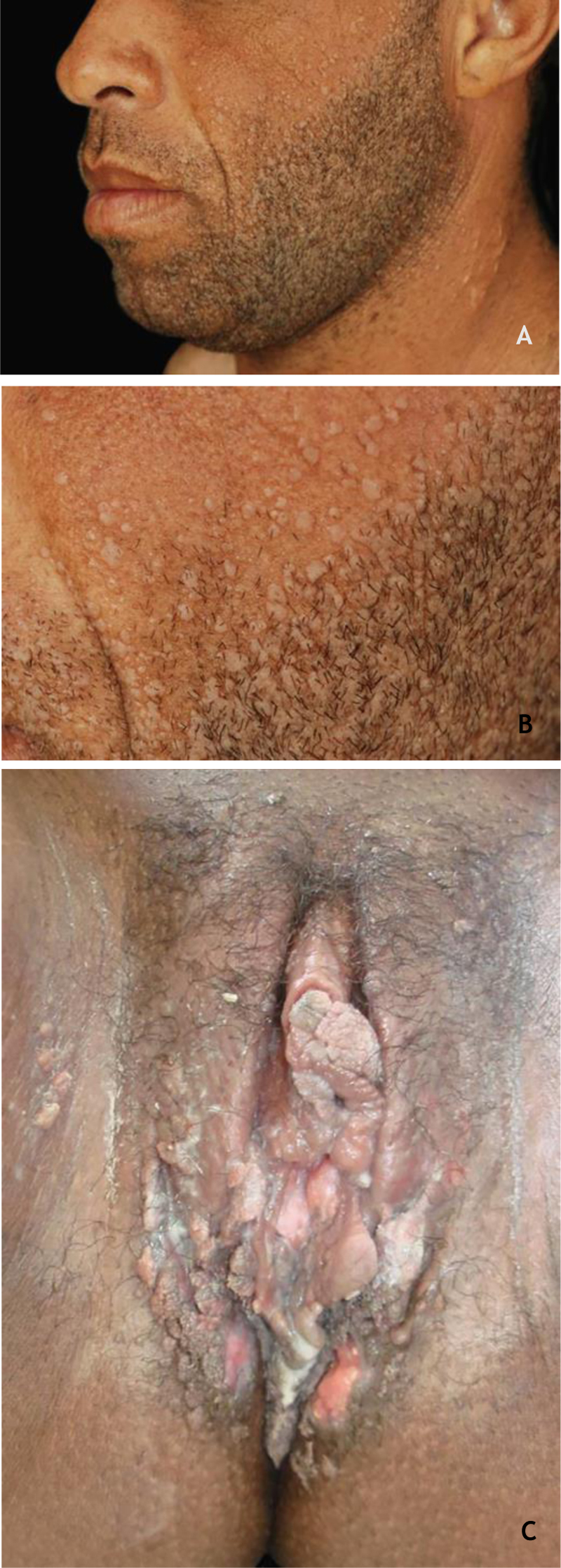
(A and B) Flat warts in a transplanted patient. (C), Condyloma acuminatum in an HIV-positive pregnant woman, showing multiple papules and condylomatous plaques, some eroded (secondary to the use of topical medication) affecting a large area. Source: HC-UFMG/EBSERH.

In renal transplant receptors (RTRs), the prevalence of CWs increases with the duration of immunosuppression; in the first year after transplantation, they are found in 15% of patients, reaching 92% after 15 years.^49^ Furthermore, the lesions are multiple, occurring in extensive areas, especially in the photoexposed regions (Fig. 9 A and B). The main types of HPV found are HPVs 2, 27, and 57.^49^ In these patients, CWs are usually refractory to treatment.^28^ Calcineurin inhibitors, such as cyclosporine, pimecrolimus, and tacrolimus, are associated with the onset of CWs, especially when associated with azathioprine. An improvement of CWs in SOT has been described when these medications are replaced by mTOR inhibitors such as sirolimus, everolimus, and temsirolimus.^50^

In HIV patients, CWs are more numerous and aggressive; they may be associated with new types of HPV, including those more typically found in the genital mucosa.^9^

### Genital warts

GWs are among the most common clinical manifestations of HPV infection in both immunosuppressed and immunocompetent individuals. HPVs 6 and 11 are the most common types found in GWs in both groups.^51^

Regarding RTR, in a prospective cohort conducted by Larsen et al. with 3,268 RTRs and 162,910 non-transplanted individuals, a three-fold greater risk of GWs was observed among the RTRs. Adjusted for age and sex, the risk was up to five times higher among RTR women.^52^

Individuals living with HIV are more susceptible to sexually transmitted infections, especially HPV. In these patients, there is a higher prevalence of co-infection with HR-HPV, especially HPV 16; the condyloma tend to be more extensive, aggressive, recurrent, and persistent^51^, ^53^ (Fig. 9C). MSM, especially HIV-positive, have a higher incidence of GWs and complications such as potentially malignant lesions and neoplasms.^37^

## The impact of HPV on the emergence of malignant neoplasms

### Non-melanoma skin cancer in immunocompetent and immunosuppressed populations

NMSC is the most frequent cancer in Brazil, corresponding to about 30% of malignant tumors registered in the country, with an estimated incidence of almost 177,000 new cases for the year 2020.^54^ Basal cell carcinoma (BCC) and SCC are the most frequent NMSC and occur mainly in areas exposed to the sun.^54^, ^55^

The relationship between β-HPVs and NMSC is controversial and has been evaluated in recent years, and is better established for people with EV and immunosuppressed patients. In immunocompetent patients, this association is weak, impaired by the heterogeneity and omnipresence of this group of viruses in overall healthy skin, which makes it difficult to define infections that may be clinically relevant.^24^, ^25^

When compared with the general population, immunocompetent patients with SCC have a higher positivity for viral DNA from at least one type of β-HPV, on the skin and/or eyebrow hair, a common reservoir of viruses of the beta genus as well as for antibodies against the viral protein L1.^56^ The main types of β-HPVs associated with the risk of SCC are: HPV 5, 8, 15, 17, 20, 24, 36, and 38.^56^

Bernat-Garcia et al. detected the presence, mainly, of β-HPVs in NMSCs and in normal perilesional skin of RTR and immunocompetent individuals. The virus was detected more frequently in the former group and in normal skin.^24^

In a review article, Sichero et al. reported a higher prevalence of β-HPVs in samples of premalignant and malignant lesions from immunosuppressed patients when compared with those immunocompetent: premalignant lesions (88% in the immunosuppressed and 54% in the immunocompetent), SCC (84% *vs*. 27%), and BCC (75% *vs*. 36%).^25^

The prevalence and viral load of β-HPV are higher in actinic keratosis, scamous dysplasia, and SCC *in situ* than in SCC proper; β-HPVs are found more frequently in eyebrow hair in patients with actinic keratoses when compared with patients with SCC. In SCC samples, no β-HPV m-RNA is observed. These findings suggest that β-HPVs would act only at an early stage of the skin carcinogenesis process and would not be necessary to maintain a mutated phenotype, as discussed in the pathogenesis.^25^

In relation to BCC, the association with HPV is even more inconclusive: the association is weaker, when compared with SCC, and there is greater divergence in the type of virus found. Ramenazi et al. conducted a systematic review of HPV infection in BCCs.^55^ Of 1,087 studies, 45 were included in the review. In seven studies, HPV assessment was performed in serum and, in 38, in BCC tissue. The findings showed a higher risk of detection of γ-HPVs in patients with BCCs when compared with healthy controls. Among α-HPVs, HPV 6 was the most frequent; among HPVs of species β1, HPV 93; and among those of species β2, HPV 23. Some limitations of that study, such as variations in HPVs, HPV typing in serum rather than in tissue, and few studies hindered the analysis of the relationship between HPV and CBC.^55^ In both immunosuppressed and immunocompetent individuals, the most frequent NMSCs are BCCs and SCCs. Infection by HPV, especially β-HPV, has been implicated in the cause of NMSC in immunosuppressed individuals, but the process of cutaneous carcinogenesis is not yet fully understood.^25^

In a meta-analysis that compared the incidence of cancer in HIV/AIDS patients with SOT, Grulich et al. found an increased frequency of NMSC in both populations, albeit higher in the transplant group.^3^

HPV DNA was found more frequently in NMSC samples in immunosuppressed (73.3%) than in immunocompetent individuals (53.3%); the most prevalent genus was β-HPV in both populations and the occurrence of different types of HPV in one lesion was greater in immunocompromised patients.^24^

In SOT, the risk of NMSC increases 40-fold in the first five years after transplantation, and may reach 100-fold throughout life.^57^ Contrary to what occurs in the general population, where BCC predominates, the most prevalent type of NMSC in SOT is SCC, in a 2–5:1 ratio.^58^ In this same group, SCC tends to manifest earlier, more exuberantly, and more aggressively.^57^

Evidence showing an association between HPV and NMSC includes the presence of warts and SCCs in patients with EV,^32^ the increased prevalence of HPV infection and NMSC among SOT,^49^, ^57^ and the occurrence of SCCs on extensive plaques of viral warts in SOT.^58^ In addition, actinic keratosis or viral warts have been associated with an increased risk of HPV in immunocompromised patients.^24^

In immunocompromised patients, the fact that skin cancer develops at an earlier age, is more aggressive, and has a greater risk of metastasis implies the need to adopt effective preventive measures for this population. Therefore, the relevance of the dermatologist in prevention is emphasized, especially with guidance on measures for photoprotection, as well as identification and treatment of pre-neoplastic lesions and neoplasms in this population.

### Carcinomas of the anogenital region in the immunosuppressed population

In Brazil, cervical cancer is the third in incidence among women.^53^ Infection with HR-HPV 16 and 18 is the main cause of this neoplasia, and immunosuppression stands out as an important risk factor.^4^, ^19^ The risk of cervical cancer is two to ten times higher in transplant recipients and five to ten times higher for women living with HIV compared to the general population.^18^ Women living with HIV have significantly higher rates of HSILs and are more susceptible to progression to invasive cervical carcinoma when compared with women without HIV.^19^

The term vulvar carcinoma encompasses both SCC of the vulva and its precursor lesions. It is estimated that about 29% to 43% of these lesions are associated with HPV infection, particularly with types 16 and 18.^4^, ^5^ VIN, currently called vulvar HSILs, designates HPV-related lesions, precursors to vulvar cancer.^38^ In immunosuppressed patients, vulvar HSILs are generally more aggressive, extensive, multifocal, and tend to recur after treatment; in these patients, invasive vulvar cancer occurs at earlier ages.^4^

Penile carcinoma is a rare entity and has a higher incidence in developing countries. HPV is responsible for approximately 50% of carcinomas in that site.^14^ SOT have an even greater risk of developing penile neoplasia, with a standardized incidence rate of 15.79 compared to 4.42 in men with HIV.^3^ Men living with HIV are two to three times more at risk of penile SCC and higher rates of PIN (precursor lesion of penile cancer associated with HR-HPV) than HIV-negative men.^4^

Anal cancer is relatively uncommon in the general population. The persistence of HR-HPV infection is responsible for 88% of anal cancers, and a significant risk factor is co-infection with HR-HPV and HIV.^20^, ^51^ The prevalence of HPV in the anogenital tract is about 60% within the sexually active population, but in HIV-positive MSM, this prevalence can reach close to 100%.^20^ The incidence of anal cancer in MSM who are HIV-positive is 80 times higher than in men in the general population and is influenced by the CD4 level of these individuals.^20^ The incidence of anal cancer is much higher among individuals living with HIV than SOT.^3^ Epidemiological studies have shown that 20% to 50% of solid organ recipients have HPV in the anal area and a ten-fold increase in the relative risk of developing anal SCC.^51^

### Oropharyngeal carcinoma in the immunosuppressed population

HPV is detected in approximately 25% of oropharyngeal cancers.^51^ This neoplasm is associated with orogenital transmission, especially HPV 16. The incidence of oral HPV infection is up to three times higher in HIV-infected individuals and is also associated with the number of oral sex partners throughout life.^51^ SOT also have a higher incidence of this type of cancer.^3^ HPV-positive oropharyngeal tumors have a better prognosis than negative ones, partly because they have a better response to treatment.^4^

In a subsequent article, histopathological aspects, complementary diagnosis, treatment, and measures for the prevention of HPV infection will be addressed, including vaccination and health education.

## Financial support

None declared.

## Authors’ contributions

Geraldo Magela Magalhães: Approval of the final version of the manuscript; design and planning of the study; drafting and editing of the manuscript; critical review of the literature; critical review of the manuscript.

Érica Cristina Vieira: Approval of the final version of the manuscript; design and planning of the study; drafting and editing of the manuscript; critical review of the literature; critical review of the manuscript.

Lucas Campos Garcia: Approval of the final version of the manuscript; design and planning of the study; drafting and editing of the manuscript; critical review of the literature; critical review of the manuscript.

Maria de Lourdes Ribeiro de Carvalho Leite: Approval of the final version of the manuscript; design and planning of the study; drafting and editing of the manuscript; critical review of the literature; critical review of the manuscript.

Antônio Carlos Martins Guedes: Approval of the final version of the manuscript; design and planning of the study; drafting and editing of the manuscript; critical review of the literature; critical review of the manuscript.

Marcelo Grossi Araújo: Approval of the final version of the manuscript; design and planning of the study; drafting and editing of the manuscript; critical review of the literature; critical review of the manuscript.

## Conflicts of interest

None declared.

ANSWERS

Donovanosis. An Bras Dermatol. 2020;95(6): 675‐683.

**Table:** 

1. b	3. b	5. c	7. a	9. b
2. a	4. a	6. d	8. c	10. a
